# Does prednisone use in pregnant women with rheumatoid arthritis induce insulin resistance in the offspring?

**DOI:** 10.1007/s10067-022-06347-0

**Published:** 2022-08-30

**Authors:** Florentien D. O. de Steenwinkel, Radboud J. E. M. Dolhain, Johanna M. W. Hazes, Anita C. S. Hokken-Koelega

**Affiliations:** 1 Department of Rheumatology, Erasmus MC, Postbus 2040, 3000 CA Rotterdam, The Netherlands; 2 Department of Paediatrics, Subdivision of Endocrinology, Erasmus MC—Sophia Children’s Hospital, Rotterdam, The Netherlands

**Keywords:** Corticosteroids, Insulin resistance, Offspring, Pregnancy

## Abstract

**Objectives:**

The use of long-term corticosteroids during pregnancy has been growing over the past decades. Corticosteroids can be given when an auto-inflammatory disease like rheumatoid arthritis (RA) is too active. Several studies have shown that long-term corticosteroids use in pregnancy is associated with maternal and fetal adverse outcomes, like preeclampsia, shorter gestational age, lower birth weight, and rapid catch-up growth. These last two outcomes could influence the insulin resistance later in life. Our objective was to investigate whether prednisone use in pregnant women with RA induces insulin resistance in offspring.

**Methods:**

One hundred three children were included after their mother had participated in a prospective cohort study on RA and pregnancy. Forty-two children were in utero exposed to prednisone and 61 were non-exposed. To assess insulin resistance, we measured homeostasis model of assessment insulin resistance (HOMA-IR) and serum adiponectin and lipid levels, corrected for body fat distribution.

**Results:**

An average of 6 mg prednisone on a daily use gave no difference in mean HOMA-IR (SD) between the children who were prednisone-exposed in utero (1.10 (0.84)) and those non-exposed (1.09 (0.49)). No difference was found in mean adiponectin level, body fat distribution, or lipid levels such as total cholesterol, fasting triglyceride, or high-density lipoprotein.

**Conclusion:**

Children who are prednisone-exposed in utero (low dose) have no increased risk for insulin resistance at the age of approximately 7 years. These findings are reassuring because the prednisone use during pregnancy is increasing worldwide. Further research has to be performed to evaluate if the insulin resistance remains absent in the future.

**Table:** 

**Key Points** *• What is already known on this topic—long-term corticosteroids use in pregnancy is associated with fetal adverse outcomes, like lower birth weight and rapid catch-up growth which can influence the insulin resistance later in life.* *• What this study adds—long-term corticosteroids use in pregnant women with rheumatoid arthritis has no increased risk for insulin resistance in the offspring.* *• How this study might affect research, practice, or policy—findings are reassuring because prednisone use during pregnancy is increasing worldwide. Further research should evaluate if the insulin resistance remains absent in the future.*

**Supplementary Information:**

The online version contains supplementary material available at 10.1007/s10067-022-06347-0.

## Introduction

The use of long-term corticosteroids during pregnancy has been growing over the past decades. Recent study showed a steady increase from 2 to 81 per 100,000 pregnancies over more than a decade [1]. Emphasizing the magnitude of this trend in a variety of immunological and inflammatory diseases, various studies show that long-term corticosteroids use in pregnancy is associated with adverse maternal and fetal adverse outcomes, like preeclampsia, lower gestational age, lower birth weight, and rapid cath-up growth [1–3]. These last two outcomes are associated with insulin resistance later in life [4].

Rheumatoid arthritis (RA) is one of those inflammatory, autoimmune diseases that is contributing to this steady increase of long-term corticosteroid use during pregnancy. It is a systemic disease which often affects women of childbearing age. RA disease activity declines in only half of the pregnancies [5]. Therefore, medication like prednisone can be critical. It is not clear to which extent prednisone can pass the placenta [6]. At least in early gestation, when the placenta has not yet fully developed, prednisone may influence the fetal programming in humans [6–8]. Fetal programming is a theory that suggests that certain actions during certain points of pregnancy may cause permanent effects on the fetus and also later in life. In fetal development, the maternal glucocorticoids are critical in normal development of the fetus, as they are involved in the growth and maturation of many organ systems [9].

Experimental studies suggested that in utero exposure to prednisone can lead to fetal programming with persistently increased glucocorticoid effects throughout childhood, creating insulin resistance later in life [7].

In healthy, young children, the Homeostasis Model of Assessment Insulin Resistance (HOMA-IR) is an appropriate ethically accepted method to measure insulin resistance [10, 11]. Serum adiponectin levels will also indicate whether children have signs of insulin resistance. Adiponectin is a hormone mainly produced by white adipose tissue and levels are significantly lower in obese people [12]. High levels of adiponectin are associated with a lower risk of myocardial infarction and development of atherosclerosis. Adiponectin plays an important role in the pathogenesis of metabolic disease, like obesity and type 2 diabetes [13–15].

The aim of our study was to explore an association between maternal prednisone use during pregnancy and the risk on insulin resistance in the offspring. We hypothesized that exposure to synthetic glucocorticoids in utero increases insulin resistance in the children. We therefore determined the insulin resistance in children who had been exposed to synthetic glucocorticoids in utero. If prednisone-exposure in utero would affect the manifestation of insulin resistance in the prepubertal offspring, it would increase the risk for a less favourable health profile later in life.

## Methods

The study cohort consisted of 103 healthy children born from mothers participating in the PARA-study (pregnancy-induced amelioration of rheumatoid arthritis). In this prospective, nationwide cohort study, pregnant women with RA (*n* = 255) were visited several times at their home address were a physical examination was performed and information on disease activity including medication use was collected [5]. To calculate the RA disease activity score (DAS28), we examined 28 joints and used 3 variables to make the calculation: number of swollen joints, number of tender joints, and serum C-reactive protein (CRP) level [16, 17]. Information on medical history regarding gestational age, birth weight, and postnatal complications were conducted. Birth weight was expressed as birth weight standard deviation scores (birth weight SDS), corrected for gestational age and gender [18].

After participating in the PARA-study, all mothers were contacted by mail and phone to participate in the follow-up study. Children aged 5 years or older were invited to visit the Sophia Children’s Hospital in Rotterdam. During this visit, fasting blood samples were taken, anthropometric measurements were performed, and a DXA-scan (dual-energy X-ray absorptiometry) to evaluate fat distribution was executed. This study has approved by the Medical Ethics Committee at the Erasmus Medical Centre (Rotterdam, The Netherlands). All parents gave their written informed consent.

### HOMA-IR

Fasting blood samples were collected between 0800 and 1000 h to determine glucose and insulin level. One child was excluded from analyses concerning insulin sensitivity due to a non-fasting blood sample. Plasma glucose was measured on a Roche modular P analyser (Roche Diagnostics, Almere, The Netherlands). Plasma insulin concentrations were measured using an Immulite 2000 (Siemens healthcare Diagnostics B.V. Den Haag, The Netherlands). For glucose, the intra- and inter-assay coefficient of variation was 0.7% and 1.2% and for insulin 5.5% and 7.3%, respectively.

Insulin resistance was calculated using the Homeostasis Model of Assessment Insulin Resistance (HOMA-IR: [insulin (μIU/mL) x glucose (mmol/L)] / 22.5) [13, 14]. Healthy children have a HOMA-IR score of 1, and insulin resistance is present when the HOMA-IR ≥ 2.5 [11, 19, 20]. Due to the skewed distribution of the HOMA-IR, analyses were performed after log-transformation.

### Adiponectin

Fasting levels of adiponectin were measured on a Roche modular P analyser (Roche Diagnostics, Almere, The Netherlands). The intra- and inter-assay coefficient of variation for samples was less than 3.82% and 5.15%.

### Fat distribution

Other factors that can contribute to the presence and development of insulin resistance are weight, more specifically body fat mass, and the distribution of the body fat. Abdominal distribution of body fat has significant impact on the development of insulin resistance and creates an increased risk for metabolic disease independent of total body fat, while fat in the lower body is protective [21–24]. We therefore measured height and weight of the child. Body mass index (BMI) was calculated by dividing weight (kilograms) by the square of height (meters).

Visceral fat distribution has significant impact on decrease in adiponectin levels, but this decrease in adiponectin levels was not seen in case of subcutaneous fat [25, 26]. Visceral fat, also known as organ fat or intra-abdominal fat, is located between internal organs and subcutaneous fat is found underneath the skin. Therefore, circumferences of the arm, waist, and hip, but also skinfolds of triceps, biceps, subscapular, and suprailiac were performed. Visceral fat distribution was assessed by the ratio waist to hip circumference and by the ratio skinfolds trunk (subscapular + suprailiac) to peripheral (triceps + biceps). All measurements were executed by the same doctor and with the same scale. Measurements were performed 3 times and the mean value was used for analysis. All values were transformed to standard deviation scores (SDS) for age and gender according to Dutch reference values [27, 28], using the Growth Analyser (version 4.0; Growth analyser BV, Rotterdam, the Netherlands, www.growthanalyser.org).

All children underwent a DXA-scan (dual-energy X-ray absorptiometry scan, type Lunar-Prodigy; GE Healthcare, Chalfont St. Giles, UK) to further describe the fat distribution of the child. The fat mass was assessed and the percentage fat mass (fat mass in grams _total body_ / fat mass in grams _total body_ + lean mass in grams _total body_) and trunk fat ratio (fat mass in grams _trunk_/ fat mass in grams _total body_) were calculated. All scans were made with the same machine, and quality assurance was performed daily. Coefficient of variation was 1.2% for the total fat mass. Fat mass and the percentage fat mass distribution were both transformed into SDS for age and gender [29–32].

### Lipid profile

Fasting levels of high-density lipoprotein (HDL), triglycerides (TG), and total cholesterol were measured on a Roche modular P analyser (Roche Diagnostics, Almere, The Netherlands), with the following intra- and inter-assay coefficient of variation: HDL, intra = 0.6% and inter = 0.9%; TG, intra = 0.7% and inter = 1.9%; total cholesterol, intra = 1.1%, and inter = 1.6%.

### Statistical analysis

The main purpose of our study was to assess the difference in insulin resistance in children with and without early exposure to prednisone in utero. Unpaired Student’s *t*-tests were performed to assess the differences between the exposed and the non-exposed group in clinical characteristics, insulin resistance (HOMA-IR and serum adiponectin), differences in lipid profiles (high-density lipoprotein, triglycerides, and total cholesterol), and differences in body fat distribution (total fat mass percentage fat, trunk fat ratio, skinfolds, and body circumference). In these analyses, prednisone use was entered as a dichotomous variable, ever use or no use.

Multivariate linear regression models were used to describe the association between HOMA-IR, plasma adiponectin, trunk fat ratio, and the maternal prednisone use during pregnancy. In these models, we added gender, age, and RA disease activity during pregnancy and also included all variables that were statistically different between the prednisone-exposed and non-exposed group. All variables were entered simultaneously in the model.

Finally, to investigate if there was a prednisone dose effect, the prednisone dose was entered as a continues variable in the third model. To determine whether the impact of the use of prednisone was greater when used early in in pregnancy, the analyses were also performed with entering prednisone use in first trimester, instead of prednisone use during the entire pregnancy. All statistical analyses were performed using STATA software (version 12.0 for Mac; StataCorp LP, Texas, USA). Statistical significance was defined as *p* < 0.05.

## Results

### Pregnancy outcome measures

Prednisone was used by 41% (42/103) of the mothers during pregnancy. Of the 42 women that used prednisone, two mothers stopped prednisone after the first trimester and five mothers started. This resulted in 83% (35/42) of the women continuing their prednisone throughout the entire pregnancy. The median prednisone dose was 6.0 mg/day (IQR: 1–15 mg). Other medication included sulfasalazine 33% (34/103) and hydroxychloroquine 2% (2/103), sometimes in combination with prednisone. The median dose of sulfasalazine was 2000 mg/day (range 500–4000 mg) (Table 1). In total, RA medication was used by 47% (48/103) of the mothers during pregnancy.

**Table 1:** Characteristics of the prednisone exposed and the non-exposed group

	Exposed (*n* = 42)	Non-exposed (*n* = 61)	
	Mean (SD)	Mean (SD)	*p*
During pregnancy			
Maternal age at delivery (years)	33.26 (3.8)	32.21 (3.81)	0.18
Maternal RA duration at delivery (years)	6.67 (5.59)	8.57 (7.06)	0.15
Maternal DAS28 first trimester Maternal DAS28 third trimester	4.26 (1.28) 3.74 (1.19)	3.25 (0.97) 3.07 (1.07)	< 0.001* < 0.01*
Maternal sulfasalazine use (Y/N)	17/25	17/44	0.18
Maternal hydroxychloroquine use (Y/N)	0 /42	2/69	0.24
At birth			
Gender (M/F)	19/23	38/23	0.09
Gestational age (weeks)	39.12 (1.89)	39.69 (1.75)	0.12
Birth weight SDS	− 0.03 (1.06)	0.11 (1.18)	0.53
Δ height 0–3 months	0.08 (0.97)	− 0.14 (0.87)	0.29
Δ weight 0–3 months	0.47 (1.08)	0.29 (0.99)	0.41
At investigation			
Age (years)	6.78 (1.14)	7.04 (1.32)	0.30
Weight (SDS)	0.26 (2.38)	− 0.05 (1.01)	0.37
Height (SDS)	− 0.07 (0.96)	0.15 (0.95)	0.25
BMI (SDS)	− 0.20 (0.84)	− 0.18 (0.90)	0.91

All data are expressed as mean (SD) or ratio (Y/N). *RA*, rheumatoid arthritis; *DAS28*, RA disease activity Score in 28 joints; *SDS*, standard deviation score; *BMI*, body mass index

* statistical significant <0.05

Mean DAS28 (SD) was significantly higher in women who used prednisone during pregnancy. In first trimester, DAS28 was 4.26 (1.28) in the prednisone-exposed group and 3.25 (0.97) in the non-exposed (*p* < 0.001). In third trimester, the RA disease activity declined in both groups to, 3.74 (1.19) and 3.07 (1.07), respectively (*p* < 0.01). The mean gestational age (SD) was 39.1 (1.89) weeks in the prednisone-expose and 39.7 (1.75) in the non-exposed (*p*- = 0.12). Mean (SD) birth weight SDS was − 0.03 (1.06) in the prednisone exposed group and 0.11 (1.18) in the non-exposed (*p* = 0.53) (Table 1). Less than 5% (4/103) of the mothers smoked during pregnancy of which only 1 mother used prednisone.

### Clinical outcome measures of children during follow-up study

At the follow-up visits of the children, the mean age (SD) in the prednisone-exposed group was 6.78 (1.14) years and in the non-exposed 7.04 (1.32) years (*p*: 0.30). The mean weight SDS, height SDS, and BMI SDS (SD) did not differ between the 2 groups (Table 1).

### Insulin resistance outcome measures

#### ***HOMA-IR***

No difference in fasting glucose, insulin levels, or HOMA-IR was observed between the children who were in utero exposed to prednisone and those who were not exposed. The mean (SD) fasting glucose level was 4.87 (0.37) mmol/L in the children exposed to prednisone and 4.94 (0.42) in the non-exposed. Fasting insulin was 4.95 (3.57) µU/mL and 4.92 (2.03) µU/mL, respectively. A total of 2.9% (3/103) of children had mild fasting hyperglycaemia (> 5.6 mmol/L) and normal fasting plasma insulin levels, but none of these children had been exposed to prednisone in utero*.* The median (SD) HOMA-IR score was 1.10 (0.84) in the children exposed to prednisone and 1.09 (0.49) in the non-exposed (Table 2). Four children had a HOMA-IR ≥ 2.5 (3.9%) of whom 3 were exposed to prednisone in utero. The prednisone dose of the 3 children who were exposed was low, 2.5, 3.5, and 4.0 mg prednisone per day, respectively. There was no difference between the presence of HOMA-IR (≥ 2.5) in the prednisone-exposed and non-exposed group (*p* = 0.16).

**Table 2:** Insulin resistance in the prednisone exposed and the non-exposed group

	Normal	Exposed (*n* = 42)	Non-exposed (*n* = 61)	
		Mean (SD)	Mean (SD)	*p*
Homeostasis model of assessment				
Fasting glucose (mmol/L)	3.0–6.5	4.87 (0.37)	4.94 (0.42)	0.34
Fasting insulin (µU/mL)	< 14	4.95 (3.57)	4.92 (2.03)	0.96
HOMA	1.00	1.10 (0.84)	1.09 (0.49)	0.99
HOMA-IR (log transformed)	0.18	0.10 (0.56)	0.18 (0.40)	0.36
Adiponectin				
Adiponectin (µg/mL)	5–15	16.45 (4.06)	15.40 (3.59)	0.17
Lipid profile				
Total cholesterol (mmol/L)	2.8–5.4	4.51 (0.79)	4.35 (0.58)	0.25
Fasting triglycerides (mmol/L)	0.3–1.1	0.85 (0.33)	0.78 (0.25)	0.29
Fasting HDL (mmol/L)	0.8–1.9	1.53 (0.27)	1.49 (0.31)	0.48
Fat distribution				
Fat mass (SDS)		0.09 (1.01)	0.26 (0.89)	0.38
Percentage fat (SDS)		0.07 (0.03)	0.07 (0.04)	0.90
Trunk fat ratio		0.36 (0.04)	0.35 (0.04)	0.39
Skinfolds				
Triceps (SDS)		− 0.25 (1.81)	− 0.06 (1.59)	0.57
Biceps (SDS)		− 0.13 (2.16)	0.18 (1.71)	0.41
Subscapular (SDS)		− 0.35 (1.70)	− 0.17 (1.84)	0.61
Suprailiac (SDS)		− 1.61 (0.98)	− 1.58 (1.25)	0.91
Ratio skinfolds trunk to peripheral		0.11 (1.76)	0.64 (1.18)	0.10
Circumferences				
Arm (SDS)		− 0.19 (1.68)	0.09 (1.51)	0.38
Waist (SDS)		0.13 (0.97)	0.18 (1.10)	0.80
Hip (SDS)		− 0.61 (1.11)	− 0.48 (0.99)	0.55
Ratio waist to hip (SDS)		1.03 (0.92)	0.96 (0.93)	0.70

All data are expressed as mean (SD). *SDS*, standard deviation score; *BMI*, body mass index; *HOMA-IR*, homeostasis model of assessment-insulin resistance; *HDL*, high-density lipoprotein

The multivariate linear regression model showed no association between maternal prednisone use during pregnancy and the HOMA-IR of the offspring. Age was associated with the HOMA-IR, *ß* = 0.12, *p* < 0.01 (Table 3).

**Table 3:** Multiple linear regression-analyses

	Adiponectin		HOMA-IR		Trunk fat ratio	
	*β*	*p*-value	*β*	*p*-value	*β*	*p*-value
Prednisone	0.50	0.55	− 0.03	0.77	0.01	0.21
RA disease activity	0.19	0.58	− 0.06	0.12	− 0.01	0.18
Age	− 0.29	0.37	0.12	< 0.01*	0.01	< 0.01*
Gender	1.70	**0.03**	0.16	0.08	0.01	0.17

*HOMA-IR*, Homeostasis Model of Assessment-Insulin Resistance; *Trunk fat ratio*, fat mass trunk/fat mass total body; *RA*, rheumatoid arthritis

* statistical significant <0.05

### Adiponectin

Children exposed to prednisone in utero had higher serum levels of adiponectin, but not significantly (*p* = 0.17). The mean (SD) adiponectin level was 16.5 (4.1) mg/L in the children exposed to prednisone and 15.4 (3.6) mg/L in the non-exposed. We did find a higher adiponectin levels in girls 16.7 (4.0) mg/L than in boys 15.1 (3.5) mg/L (*p* < 0.03).

The multivariate linear regression model showed no association between maternal prednisone use during pregnancy and serum adiponectin levels of the offspring. Gender was again associated with the adiponectin level, *ß* = 1.70, *p* < 0.03 (Table 3), indicating that girls have higher adiponectin levels than boys.

### Fat distribution

No difference in weight, height, or fat distribution including total fat mass, percentage fat, trunk fat ratio, skinfolds, or body circumference was observed between the children who were in utero exposed to prednisone and the children who were not exposed. The mean fat mass SDS (SD) was 0.09 (1.01) in the prednisone-exposed children and 0.26 (0.89) in the non-exposed. The trunk fat ratio was 0.36 (0.04) and 0.35 (0.04), respectively. The ratio skinfolds trunk (subscapular + suprailiac) to peripheral (triceps + biceps) was 0.11 (1.76) in the prednisone-exposed children and 0.64 (1.18) in the non-exposed (Table 2).

Skinfolds were all within the normal range and similar to the reference mean, except for the suprailiac skinfold, which was significantly lower than the mean of healthy controls (− 1.61 (0.98) SDS, *p* < 0.001) (Table 2). Circumference of the arm, waist, and hip was also all within the normal range and similar to the reference mean. The waist to hip ratio (SD) was 1.03 (0.92) in the prednisone-exposed children and 0.96 (0.93) in the non-exposed (Table 2).

The multivariate linear regression model showed no association between maternal prednisone use during pregnancy and trunk fat ratio of the offspring. Age was associated with the trunk fat ratio (*ß* = 0.01, *p* < 0.01) (Table 3).

### Other outcome measures

#### Lipid profile

The mean total cholesterol (SD) was 4.51 (0.79) mmol/L in the prednisone-exposed group and 4.35 (0.58) mmol/L in the non-exposed. Also, the fasting triglyceride level and HDL level did not differ in the prednisone-exposed or non-exposed group (Table 2).

#### Prednisone dose

In the final models in Table 3, prednisone use during pregnancy was entered as a dichotomous variable (yes/no). When we entered the prednisone medication dose as a continues variable, no association was present between the HOMA-IR (*p* = 0.81), adiponectin level (*p* = 0.50), or trunk fat ratio (*p* = 0.56). We therefore concluded that there was no prednisone dose effect. It was not possible to investigate if there was a difference between prednisone use only in first trimester and prednisone use during the entire pregnancy because 84% of the women continued their prednisone throughout the entire pregnancy. Only two women stopped their prednisone after the first trimester and five started prednisone after the first trimester.

## Discussion

Children born from mothers with RA and in utero exposed to prednisone had no signs of insulin resistance at the age of approximately 7 years. Our hypothesis that prednisone-exposure in the first trimester of pregnancy is associated with early determinants of insulin resistance in the offspring is therefore refuted. This conclusion is reassuring because the prednisone use during pregnancy is increasing worldwide not only in patients with RA.

### Interpretation of the date

Our conclusion is mainly based on the HOMA-IR, which was similar between the exposed and non-exposed. Furthermore, both groups did not differ significantly from 1.00, which is the normal value for the HOMA-IR. HOMA-IR is an appropriate method to measure insulin resistance among healthy children. The method is preferred above other fasting methods like the fasting glucose/insulin ratio (FGIR) or quantitative insulin sensitivity check index (QUICKI) [10, 11]. In line with literature, the HOMA-IR logscore was positively associated with the BMI of the child [33].

In our population, the children who were in utero exposed to synthetic glucocorticoids had similar adiponectin levels than the non-exposed. Adiponectin is mainly secreted by white adipose tissue, but expression can also be found in bone marrow, osteoblasts, and fetal tissue [34]. Circulating adiponectin concentration can be regulated by various hormonal, nutritional, and pharmacological factors, but can be downregulated by prolactin, growth hormone, and glucocorticoids [35, 36]. Furthermore, pro-inflammatory cytokines such as tumor necrosis factor α (TNFα) and interleukin-6 (IL-6) are potent inhibitors of adiponectin gene expression [36]. Adiponectin plays a role in fetal growth. In the beginning of pregnancy, maternal adiponectin levels increase and later in pregnancy the levels decrease proportionally to weight gain and the physiological decline in insulin resistance [34].

In our study population, we found a gender difference in adiponectin plasma concentration. The mean adiponectin level was higher in girls than in boys. Literature described this difference, although it was reported that the difference usually starts after puberty [37, 38]. Even though our study population was at prepubertal age, we had the same observation of a significant higher adiponectin level in the girls. Gender was also the only significant determinant of adiponectin in the multivariate analyses.

Glucocorticoids regulate multiple steps in the process of adipogenesis. They play an important role in the development of obesity, particularity fat deposits in the trunk and the pathogenesis of obesity related diseases [22]. Consequently, when investigating insulin resistance, the trunk fat ratio needs to be taken into account. Children exposed in utero to prednisone had similar body fat percentage and fat distribution compared to children without prednisone-exposure, and compared to the normal population. These findings are reassuring, because these variables have a great predictive value on the fat mass throughout adulthood [39, 40].

### Strength and limitations

The main strength of this study is that all pregnancies were prospectively followed. Medication intake and RA disease activity was followed throughout the entire pregnancy and always assessed by the same research assistants. Also all data concerning the prepubertal children were measured and collected by one doctor to preserve the continuity. Although only 54% of all eligible children participated, there was no statistical difference between the participating and non-participating group (Supplementary Table). We conducted a nationwide study, but all children had to be seen Sophia Children’s Hospital in Rotterdam. Main reason for non-participation was the distance to the hospital (38%). Other reason was that parents felt that our investigations were too much of a burden for their child (30%). Another limitation could be that no hyperinsulinemic euglycemic clamp (HEC), intravenous, or oral glucose tolerance test (OGTT) was used to quantify the insulin resistance. Taking our objective, we used the less-invasive and therefore ethically approved HOMA-IR calculation to assess the insulin resistances in this healthy study population between 5 and 10 years old [10]. Several studies have shown a reasonable correlation between HOMA-IR levels and results from HEC measurements or OGTT [41–44].

Use of prednisone during pregnancy has been rapidly growing over the past decade [1]. This use is associated with maternal and fetal adverse outcomes. The aim of this study was to explore an association between prednisone use during early pregnancy and the risk of insulin resistance later in life of the offspring. We can now conclude that children who are exposed to “low dose” of synthetic glucocorticoids in utero have no higher risk for insulin resistance at the age of approximately 7 years. Nevertheless, further research has to be performed to evaluate if these risk factors remain absent in the future.

## Supplementary Information

Below is the link to the electronic supplementary material.

Supplementary file1 (DOCX 36 KB)
