# Supplementary material for: Does prednisone use in pregnant women with rheumatoid arthritis induce insulin resistance in the offspring?
Source: Clin Rheumatol. 2022 Aug 30;42(1):47–54. doi: 10.1007/s10067-022-06347-0 (PMC9823030; doi:10.1007/s10067-022-06347-0)
Supplement: Supplementary file 1 — Supplementary file1 (DOCX 36 KB) [file 10067_2022_6347_MOESM1_ESM.docx]

| ***Supplementary Table -*** *Mother and Child Characteristics of the population studied and those lost-to-follow-up* | | | |
| --- | --- | --- | --- |
|  | *Study population*  ***(n=103)*** | *non-participating group*  ***(n=88)**** |  |
| ***Clinical Characteristics Mother*** |  |  | ***p*** |
| *Age at delivery (years)* | *32.6 (3.81)* | *32.3 (3.55)^a^* | *0.63* |
| *RA duration at delivery (years)* | *7.49 (6.33)* | *6.29 (6.08)* | *0.19* |
|  |  |  |  |
| ***Disease activity during pregnancy (DAS28)*** |  |  |  |
| *trimester 1*  *trimester 3* | *3.65 (1.19)*  *3.33 (1.18)* | *3.70 (1.03)*  *3.35(1.04)* | *0.77*  *0.88* |
| ***Use of Medication during pregnancy*** |  |  | ***p*** |
| *No medication*  *Prednisone*  *Only Sulfasalazine* | *44 (42)*  *26 (25)*  *17 (16)* | *41 (48)*  *24 (28)*  *14(16)* | *0.33* |
| *Combination of two* | *18 (17)* | *7 (8)* |  |
|  |  |  |  |
| *Smoking during pregnancy* | *5(5)* | *9 (10)* | *0.13* |
|  |  |  |  |
| ***Clinical Characteristics Child*** | ***(n=103)*** | ***(n=88)*** | ***p*** |
| *Birth weight (kilograms)* | *3.422* | *3.255^a^* | *0.06* |
| *Gestational age (weeks)* | *39.5* | *39.1^a^* | *0.22* |
| *Birth weight SDS* | *0.05* | *-0.20^a^* | *0.11* |
| *Gender (M/F)* | *57/46* | *51/34^a^* | *0.43* |
| ***Mode of feeding 12 weeks post-partum*** |  |  | ***p*** |
| *Only breast*  *Only formula*  *Both* | *34 (35/103)*  *61 (63/103)*  *5 (5/103)* | *40 (34/85)*  *53 (45/85)*  *7 (6/85)* | *0.50* |
| *All data are expressed as mean (SD) or percentage * lost-to-follow-up (n=13) + unwilling to participate (n=75) ; ^a^ n =85; Abbreviations: RA, rheumatoid arthritis; DAS28, RA Disease Activity Score in 28 joints with CRP levels; SDS, Standard Deviation Score* | | | |

***Supplementary* Figure** : Flowchart of the population studied

**Born alive in the PARA study**

n = 255

**Excluded for present analyses (n = 64)**

Less than 5 years old n = 57

No blood samples of child n = 4

Diabetes Mellitus type I of child n = 1

No fasting levels of child n = 1

Breast cancer during pregnancy n = 1

**Eligible for present analyses**

n = 191

**Data not available for present analyses (n = 88)**

Unwilling to Participate n = 75

Lost to follow up n = 13

**Data available for analyses**

n = 103
